# Induced feelings of external influence during instructed imaginations in healthy subjects

**DOI:** 10.3389/fpsyg.2022.1005479

**Published:** 2022-11-01

**Authors:** Kathrin N. Eckstein, David Rosenbaum, Nadine Zehender, Sonja Pleiss, Sharon Platzbecker, Anne Martinelli, Matthias L. Herrmann, Dirk Wildgruber

**Affiliations:** ^1^Department of Psychiatry and Psychotherapy, Tübingen Center for Mental Health (TüCMH), University of Tübingen, Tübingen, Germany; ^2^Department of Psychiatry and Psychotherapy, University Medical Center, Faculty of Medicine, University of Freiburg, Freiburg, Germany; ^3^School of Psychology, Fresenius University of Applied Sciences, Frankfurt am Main, Germany; ^4^Department of Neurology and Neuroscience, Medical Center, University of Freiburg, Freiburg, Germany

**Keywords:** delusion of influence, psychotic symptoms, sense of agency, emotional valence, response latency, tDCS, eye contact, hand touch

## Abstract

The psychopathological phenomenon of delusions of influence comprises variable disturbances of the self-environment-border leading to the feeling of external influence on thoughts, feelings, impulses or behaviors. Delusions of influence are a hallmark in psychotic illness, but nevertheless, attenuated forms can also appear in healthy individuals. Here we present a newly developed paradigm to induce and assess feelings of external influence during instructed imaginations in healthy individuals. In the current study, we asked 60 healthy individuals to visually imagine different objects. To induce feelings of external influence, we applied one of three different physical setups (low-amplitude transcranial direct current stimulation, eye contact, or skin-to-skin hand touch), and informed the participants whether or not an external influence was attempted during the respective trial. The physical setup (setup vs. no setup, *Z* = −3.847, *p* < 0.001, *r* = 0.497) as well as the information given to the participants (confirmation vs. negation, *Z* = −5.218, *p* < 0.001, *r* = 0.674) alone were able to modulate the feeling of external influence in all three interventions. The impact of information (whether influence was attempted or not attempted) significantly exceeded the impact of the physical setup on the ratings of experienced external influence (*Z* = −2.394, *p* = 0.016, *r* = 0.310). Moreover, the response latency correlated with the estimated feeling of external influence (*r*_S_ = 0.392, *p* = 0.002). Additional analyses addressed the influence of the emotional content of imagined objects and examined the intensity and emotional valence of the imaginations. Further supplemental analyses correlated external influence estimation of the participants with other psychopathological measures (trait markers for supernatural beliefs, proneness to hallucinations, and delusions and attributional style). In conclusion, this study endorses a quantitative model of psychopathological characteristics, in this case feelings of external influence that can be induced by external cues.

## Introduction

Delusions of influence describe a diverse group of disturbances. They include delusions of control, i.e., the (false) belief of external influence on thoughts, feelings, impulses, or behaviors, as also manipulations of thoughts, such as thought insertion, withdrawal, and broadcasting (Broome et al., 2018). It is assumed, that this psychopathological complex is caused by disturbances in the self-other-border differentiating between self-and other-generated stimuli. Previous investigations found this complex as a distinct factor of psychotic syndromes (Kimhy et al., 2005). Delusions of influence build a major complex in schizophrenic symptomatology (Blakemore et al., 2000; Frith et al., 2000a,b) and play a crucial role for diagnostic identification of schizophrenic disorders (Dilling, 2015) and for differentiation from other diagnoses, non-psychotic mental health disorders and other types of psychosis (Soares-Weiser et al., 2015).

The emphasis of delusions of influence in theoretical models of psychosis seems justified, as several crucial clinical and neurobiological features are associated with the presence of such self-disturbances. First, the presence of these phenomena is a good predictor of an imminent onset of illness in high risk individuals (Klosterkotter et al., 2001; Parnas et al., 2011; Nelson et al., 2012). Second, the severity of delusions of influence at the initial manifestation differentiates between patients with schizophrenia and patients with bipolar disorders and other psychotic illnesses (Haug et al., 2012a). Third, the severity of these symptoms correlates with the level of social functioning (Haug et al., 2014) and with suicidality (Skodlar and Parnas, 2010; Haug et al., 2012b). Finally, the different facets of delusions of influence build a stable network structure over time (Raballo and Preti, 2018). The symptoms of delusions of influence show high persistence rates over 5 years of duration of illness (Nordgaard et al., 2017, 2018). They are not just a manifestation of impaired cognitive function, as Nordgaard and Parnas (2014) showed that delusions of influence are not correlated with intelligence in a group of first admitted patients with non-affective psychosis (Nordgaard and Parnas, 2014).

It is noteworthy, that attenuated forms of delusions of influence, those not fulfilling all criteria, can be described as feelings of external influence, and exist also in healthy subjects. In a large representative worldwide cohort of 31,261 healthy participants lifetime prevalence of delusional experience was reported in 1.3% of cases, specifically: thought insertion and withdrawal 0.4%, mind control and/or passivity 0.3%, ideas of reference 0.4%, and plot to harm and/or follow 0.7% (McGrath et al., 2015). There may be a transition of subthreshold psychotic experiences to manifest psychotic disorders, as a stable dose–response effect has been shown in a large meta-analysis: Exposure severity (certainty of symptom, frequency of symptom, number of symptoms, persistence over time, and co-morbid depression) correlated with the risk of transition to psychotic clinical outcome in population-based samples (Kaymaz et al., 2012). These results denote a continuum of psychotic proneness.

Delusions of influence have been mainly assessed using questionnaires (Lindner et al., 2005; Synofzik et al., 2010; Haug et al., 2014; Nordgaard et al., 2017, 2018). Only few studies used special experimental designs to directly examine and modulate the feeling of external influence on thoughts [to what extend authorship of thoughts is attributed to an external entity]. Klock et al. (2021) instructed healthy participants that transcranial magnetic stimulation (TMS) of the frontal cortex could trigger the thought of an animal (similar to the generation of an involuntary movement stimulating the motor cortex). They informed the participants that they indicate by color, whether the TMS device would be (1) active or (2) not active or (3) in an ambiguous condition the participant was not informed about the activity of the device. But indeed, the TMS device was deactivated throughout the entire experiment. The participants were asked to think about an animal. After each trial the participants answered the question, to what extend they had the feeling that the thought was produced by themselves or influenced by the external device on a visual analogue scale ranging from “not at all/TMS” to “very sure/Me.” Thus, the authors asked the participants to attribute their feelings on a dimensional scale ranging from “feeling of external influence” to “feeling of own authorship.” In our view, however, “feelings of external influence” and “feelings of own authorship” do not mandatorily have to be directly associated at the conceptual level. In principle it could be possible that they could vary independently from each other in specific situations (e.g., in specific situations a strong feeling of external influence might be paralleled by a strong feeling of (partial) own authorship, whereas in other situations there might be a complete lack of any feeling or attribution of authorship at all).

In the current work, we established a paradigm solely focused to induce and assess feelings of external influence in healthy participants as a model for delusions of influence based on a mental imagination task. Moreover, we designed different conditions to differentiate between the impact of the experimental setting and the information explicitly given to the participants. Specific mental imagination procedures have been long established, initially mainly in the field of philosophy (MacKisack et al., 2016). Imagination comprises the ability to simulate objects and sensations in the mind in the absence of a corresponding sensory stimulation (Pearson et al., 2015). Mental imagery covers all five senses, but predominantly visual imagery was assessed. Nevertheless, even within one sense modality, underlying tasks were quite diverse reaching from the imagination of (previously shown) colors or geometrical shapes to imagination of movements, and results are therefore difficult to compare (Pearson, 2019).

In the present study, participants were asked to visually imagine different everyday life objects. During the imagination we aimed to modulate the experience of external influence by applying different interventions. It has been shown before that external stimuli and the information given to the participants can modulate the feeling of influence (Klock et al., 2021). Thus, we constructed interventions to examine these two components separately: (1) A physical setup: either very low amplitude (0.4 mA) transcranial direct current stimulation (tDCS), eye contact, skin-to-skin hand touch. (2) An informational component: participants were informed about whether an attempt of external influence was made in the current trial or not. The following three hypotheses were evaluated:

- The intervention (consisting of the two components physical setup and information of attempted influence) has an impact on the estimation of external influence during instructed imaginations.
- The presence of the physical setup (e.g., tDCS device, direct eye contact, or skin-to-skin hand contact) augments the feeling of external influence in comparison to the condition without the setup, even if the attempt to influence the imagination is explicitly negated.
- The information of an attempted influence (confirmation vs. negation) elevates the feeling of external influence.

Furthermore, explorative analyses tested whether the impact of information or the impact of the physical setup outweighs in the estimation of external influence, and whether not only the subjective feeling of external influence, but also the response latency required to make this judgment is sensitive to differences in the physical setup or informational components.

## Materials and methods

### Participants

60 healthy participants with normal or corrected-to-normal vision and hearing were recruited from the degree program “Psychology” of the University of Tübingen. The experiments were performed at the Department of Psychiatry and Psychotherapy, University hospital Tübingen. Inclusion criteria comprised age between 18 and 65 years and sufficient knowledge of the German language. Exclusion criteria were psychiatric disorders [assessed by Ackenheil et al. (1999)], intellectual disability or known structural brain abnormalities. None of the participants took psychopharmacological medication. Participants were randomly allocated to three different interventions, differing in the implemented physical setup.

The examination included questionnaires for proneness to hallucinations [Launay-Slade hallucination scale—revised version, LSHS-R (Launay, 1981; Bentall, 1985; Lincoln and Rief, 2009)], supernatural beliefs [Supernatural Belief Scale, SBS (Jong and Halberstadt, 2013)], absorption [Tellegen Absorption Scale, TAS (Tellegen and Atkinson, 1974; Ritz and Dahme, 1995)], attributional style [Attributionsstilfragebogen für Erwachsene, ASF-E (Poppe and Pelster, 2005)], and estimated crystallized intelligence [measured with Multiple-choice Vocabulary Intelligence Test, MWT-B (Lehrl, 2005), values given as intelligence quotient (IQ)].

A chi-square test was used to compare gender and educational level between the groups. As expected cell frequencies were below five so the exact Fisher test with Monte-Carlo significance was added. For all interval scaled variables we applied a one-way ANOVA. Due to non-normally distributed data we added nonparametric post-hoc testing with Bonferroni correction.

### Ethics statement

The study was planned and performed in accordance with the ethical principles of the Declaration of Helsinki (Code of Ethics of the World Medical Association) and was approved by the Ethics Committee of the Faculty of Medicine of the Eberhard Karls University and the University Hospital Tübingen (Ethical Approval number 018/2017BO2). Written informed consent was obtained from all participants prior to inclusion in the study. They received a small financial compensation for their participation (10 Euro per hour). Following the experiment, the participants were informed that no direct influence of the intervention on the imagination is to be expected and another written consent for further usage of the data was obtained (post-interventional informed consent).

### Stimulus material, task, and procedure

**Figure 1:**
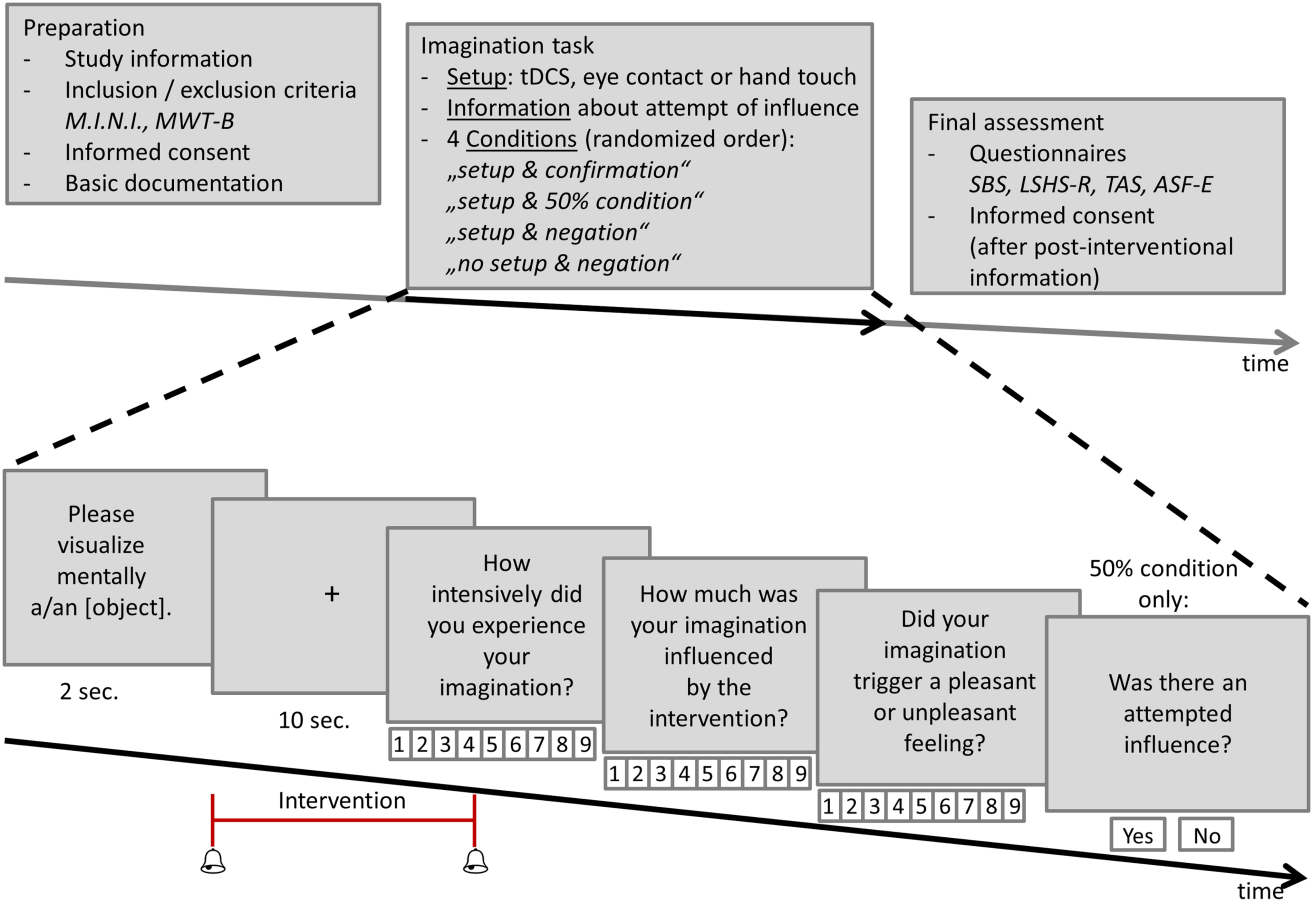
Experimental design. Upper row: Following study preparation, the physical setup was prepared (tDCS, eye contact, or hand touch) and participants were informed whether external influence was attempted in the next trial block (confirmation/negation/50% condition). Following the completion of the imagination task, the final assessments were carried out (including a post-interventional informed consent). Lower row: The imagination task started with the request to mentally visualize an object for 10 s. The beginning and the end of the imagination period was signaled by a short sound. During this imagination the intervention was carried out according to the respective, randomized condition comprising setup (tDCS, eye contact, or hand touch) and information on whether external influence was attempted (confirmation/negation/50% condition). Afterwards the participants were asked to rate the intensity of their imagination, the extent of perceived external influence and the emotional valence of their imagination on a 9-point Likert scale ranging from 1 = very low/unpleasant to 9 = very high/pleasant. In the setup and 50% condition an additional question was asked concerning the assumption of whether external influence was attempted in the last trial.

Participants were recruited via email and online posting using social networks. All experiments took place in the same examination room with a table, laptop, and two chairs in the middle of the room. Apart from that, the room was equipped with office furniture, but was otherwise empty. The investigator explained the study procedure. First, participants were informed orally and in writing. Inclusion and exclusion criteria were checked. Written informed consent was obtained from all participants (Figure 1 upper row, left side, “Preparation”).

Afterwards, the imagination task was performed (Figure 1 upper row, middle, “Imagination task,” and time sequence in the lower row). All stimuli were presented on a laptop with the program Presentation (Neurobehavioral Systems Inc., Berkeley, CA, USA). All descriptions were given visually presented on the screen. The experiment started with the instruction that the experiment aimed to assess the subjective feelings of external influence on instructed imaginations during specific external interventions. Therefore, the participants were asked to visually imagine objects from different categories, e.g., a balloon. The external interventions consisted of two components, i.e., (1) of a physical setup and (2) of an information, i.e., the participants were informed whether an influence was attempted in the current trial block or not. The participants were randomly assigned to one of the following three setups.

1. Low-amplitude transcranial direct current stimulation (tDCS) with a tDCS device (neuroConn DC STIMULATOR, neuroCare Group GmbH, Ilmenau, Germany). The anode electrode was placed over F3 according to the 10/20 EEG positioning reference, the cathode electrode on the right upper arm. After skin cleansing, the 5×7 cm electrodes were attached using electrode gel (ten20 Conductive Neurodiagnostic Electrode Paste, Weaver and Company, Aurora, CO, USA). The resistance was below 10 kOhm. The stimulation was applied during the 10 s of imagination, with additional 5 s for fade-in and fade-out, respectively. The amplitude was set to 0.4 mA, which is assumed to be below behavioral efficacy (Antal et al., 2017). In an analogous manner, sham stimulation was applied. Here, currents were applicated only during the short fade-in and fade-out phases, but no current flowed during the actual stimulation period.
2. Eye contact with neutral facial expression of an unknown person wearing a white doctor’s coat (during the 10 s of imagination). Participants were instructed to hold eye contact during this time.
3. Skin-to-skin hand touch of the dry and warm (between 30°C and 34°C measured with a surface temperature thermometer) palm of the hand of an unknown person wearing a white doctor’s coat with the self-weight of the hand touching the back of the participant’s hand (during the 10 s of imagination). The participants were instructed to look at the fixation cross, the staff member sideways past the participant.

For eye contact and hand touch a—to the participant unknown—staff member wearing a white doctor’s coat was introduced as a person especially experienced in the technique of influencing imaginations by hand touch and eye contact, respectively. Staff members were not actually trained in influencing imaginations in any manner. However, they did attempt to influence the imaginations during the “attempt” trials (see below) on a mental basis without physical engagement. The attempted influence was therefore intended as an immaterial, exclusively mental effort. For the eye contact condition the staff member sat opposite the participant, for the hand touch condition beside the participant. Between the interventions the staff member turned sideways. Altogether four male staff members acted as influencers. For the tDCS condition we performed a 10 s stimulation period (during the imagination) as attempt of influence, whereas sham stimulation with no stimulation during the 10 s served as no attempt trial. The operation of the tDCS device was performed by the investigator outside the field of vision of the participant. Pictures of the experimental arrangement can be found in Supplementary Figure 1.

Taking the two components setup and information together, every participant experienced four different conditions. The sequence of the four conditions was randomized.

1. “setup & confirmation”: The setup was applied (i.e., connected tDCS device, eye contact, or hand touch) and the participant was informed that an influence was attempted. For the tDCS condition a verum stimulation was applied. For eye contact and hand touch, the staff members did not attempt to influence the participant’s imagination.
2. “setup & negation”: The setup was applied and the participant was informed that no influence was attempted. For the tDCS condition a sham stimulation was applied. For eye contact and hand touch, the staff members did not attempt to influence the participant’s imagination.
3. “no setup & negation”: No external interventional setup was applied (i.e., no connected tDCS device, no eye contact or no hand touch) and the participant was informed that no influence was attempted.
4. “setup & 50% condition”: The setup was applied (connected tDCS device, eye contact, or hand touch) and participants were informed that an influence would be attempted in 50% of trials without knowing on which ones). In the further analysis these trials were separately divided according to:
  a) whether an influence was attempted or not (“attempt”:tDCS verum stimulation, staff members attempted to influence the participant’s imagination; “no attempt”: tDCS sham stimulation, staff members did not attempt to influence the participant), and
  b) whether the participant assumed an influence in the current trial or not (“assumption” and “no assumption”) according to the participant’s answer to the question “Was there an attempted influence?.”

Altogether 60 runs with different objects were performed. The conditions 1) to 3) were performed in 12 trials each, condition 4) was performed in 24 trials – with attempt and no attempt of influence in 12 trials each. The objects were grouped into three categories: 20 “general” objects (e.g., animal), 20 “specific positive” objects with a more positive connotation (e.g., rabbit), and 20 “specific negative” objects with a more negative connotation (e.g., spider) were presented. The list of the 60 objects can be found in Supplementary Table 2. The allocation of objects to the different conditions was pseudo-randomized, so that “general,” “specific positive,” and “specific negative” objects were equally distributed among different conditions and pseudo-randomized in their order of appearance, so the same category was never presented twice in a row.

The procedure of the imagination task (Figure 1 lower row) started with the request to visually imagine an object (presented for 2 seconds), followed by a fixation cross for the imagination (presented for 10 seconds). A short sound additionally indicated the beginning and the end of the imagination period. After each imagination the participants were asked for the intensity of the imagination, the external influence on their imagination and the emotional valence of their imagination on a 9-point Likert scale ranging from 1 = very low/unpleasant to 9 = very high/pleasant. Under the “setup & 50% condition” participants were additionally asked about their assumption of attempted influence in the current trial.

After completion of the imagination task the participants were asked by the investigator to complete questionnaires. They were informed that no direct influence of the intervention on the imagination is to be expected. For this post-interventional information another written consent for further usage of the data was obtained (Figure 1 upper row, right side, “Final assessment”).

### Data analysis

The data were analyzed with the software IBM SPSS Statistics Version 28 (IBM Corporation, Armonk, NY, USA). The values for intensity, external influence and emotional valence of the imagination as well as the response latency were recorded. The parameters did not follow a Gaussian distribution (all assessed by Shapiro–Wilk test with *p* < 0.05). To evaluate our hypothesis of a modulation of the estimated external influence by the different conditions, we performed mixed ANOVAs with a Greenhouse–Geisser correction. In all ANOVAs, the condition was included as within-subject factor and the setup (tDCS, eye contact, and hand touch) as between-subject factor. We performed four separate analyses: (1) for the conditions “setup & confirmation,” “setup & 50% condition,” “setup & negation,” and “no setup & negation,” (2) for the “impact of information” (difference between “setup & confirmation” and “setup & negation”) vs. “impact of setup” (difference between “setup & negation” and “no setup & negation”), and finally (3) two analyses for the “setup & 50% condition” (2a) divided into trials with “attempt” and “no attempt” of influence, (2b) divided into trials with “assumption” and “no assumption” of influence (according to the answer to the question “Was there an attempted influence?”). To account for the problem of non-normally distributed data we added tests for nonparametric data for all non-normally distributed datasets (for related samples using the Friedman test and the Wilcoxon signed rank test). Effect sizes of median values were calculated using *r* (*r* = |*Z*/ √n |; Rosenthal, 1991). *R* values <0.3 indicate a small effect, between 0.3 and 0.5 an intermediate effect, and r values >0.5 a strong effect. To account for multiple testing, *p*-values were corrected using the Benjamini-Hochberg procedure (FDR, false discovery rate).

Under the “setup & 50% condition,” the accuracy of a correct identification, whether an influence was attempted in the current trial was compared to chance level using a two-sided one sample t-test with a hypothesized value of 0.5. Correlational analyses were applied between the mean estimated external influence (across all conditions) of each participant and the corresponding response latency using two-sided Spearman’s correlation coefficient for non-normally distributed data. *p* < 0.05 was considered to be significant. Additional analyses compared the mean response latencies during the different conditions applying a mixed ANOVA with Greenhouse–Geisser correction and nonparametric post-hoc testing as described above.

Further exploratory analyses reported in the Supplementary Material section comprised the following procedures: The influence of the given object category (general, specific positive, specific negative) on estimated external influence, intensity and emotional valence of the imaginations was addressed in three separate mixed ANOVAs with Greenhouse–Geisser correction. To analyze the effects on intensity and emotional valence of the imaginations, two mixed ANOVAs with Greenhouse–Geisser correction were performed and nonparametric post-hoc testing added as described above. Finally, the following supplemental correlational analyses were applied (1) between mean external influence and intensity and emotional valence across all conditions, and (2) between the mean estimated external influence of each participant across all conditions and the questionnaire results for LSHS-R, SBS, TAS, ASF-E and MWT-B using Spearman’s correlation coefficient for non-normally distributed data. Two-sided tests were applied. *p* < 0.05 was considered to be significant.

## Results

### Participants’ sample description

Table 1 shows the participants’ socio-demographic characteristics and Supplementary Table 1 the psychometric data, including *p* values of the statistical analyses.

**Table 1:** Socio-demographic data.

	tDCS (*n* = 20)	Eye contact (*n* = 20)	Hand touch (*n* = 20)	*p* value
Age (years)	21.6 ± 4.3	22.0 ± 3.3	21.0 ± 2.1	0.911
Gender (m/f)	4/16	4/16	5/15	1.000
Educational level	High school graduation (*n* = 19), secondary school certificate (*n* = 1)	High school graduation (*n* = 20)	High school graduation (*n* = 20)	1.000

tDCS, transcranial direct current stimulation; m, male, f, female. Numbers indicate mean and standard deviation.

There were no significant differences in age, gender, and educational level among the three groups [all *F* (2, 57) ≤ 1.00, all *p* ≥ 0.911].

### External influence estimation during the intervention

There was a statistically significant main effect of the condition (“setup & confirmation,” “setup & 50% condition,” “setup & negation,” “no setup & negation”) on the estimation of external influence [*F*(2.207, 125.816) = 34.225, *p* < 0.001, partial *ɳ*_p_^2^ = 0.375].

**Figure 2:**
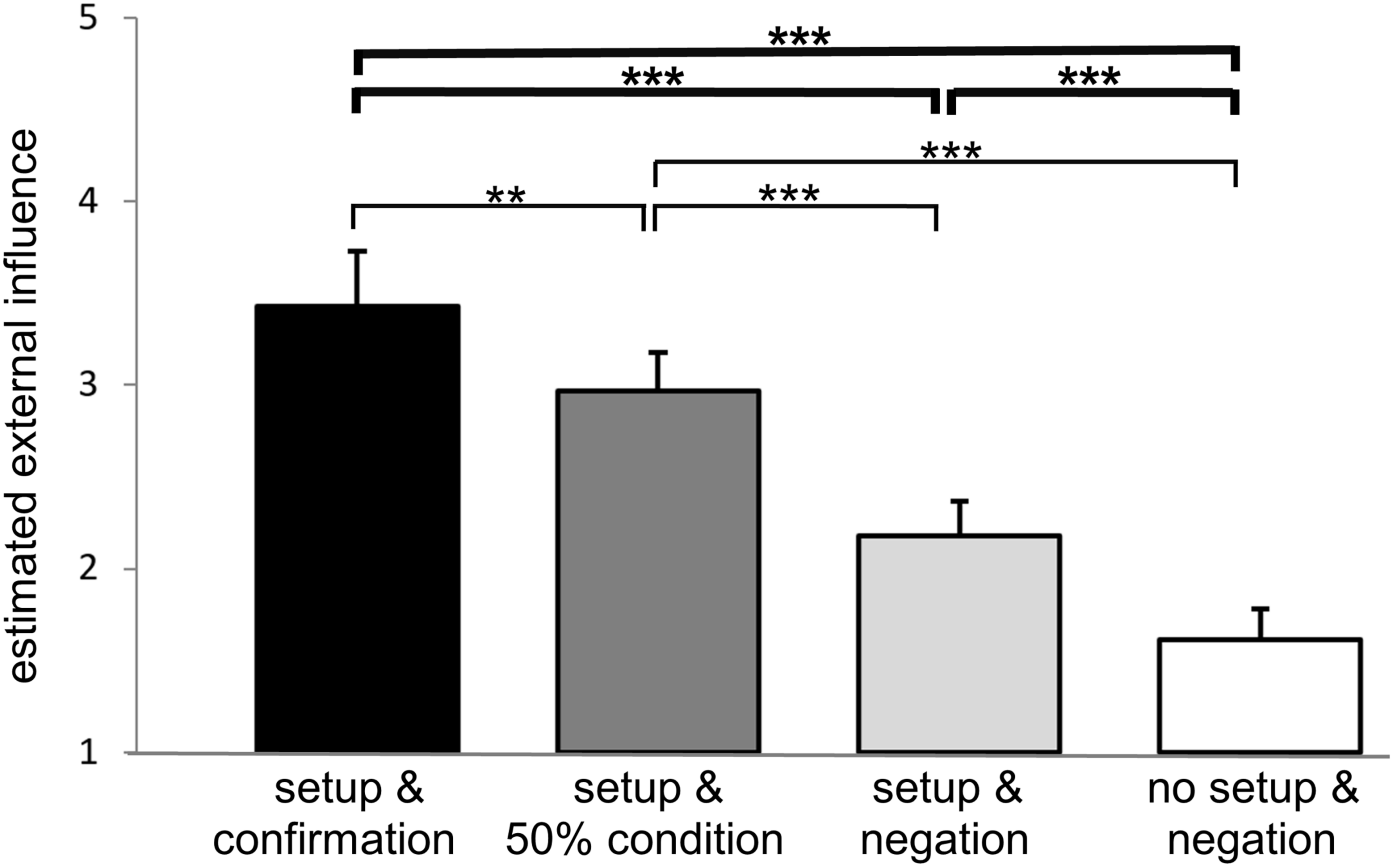
External influence estimation. Mean estimated external influence ratings of the participants for the four conditions tested are given in bars: “setup & confirmation” (black), “setup & 50% condition” (dark grey), “setup & negation” (light grey) and “no setup & negation” (white). Results of hypothesis driven analyses are marked with bold lines and asterisks above, exploratory analyses in slim lines and asterisks below. The intervention comprising setup and information has a significant impact on the estimation of external influence. Moreover, each of the two components, the setup as well as the information, contribute significantly to an increase of perceived external influence. Furthermore, exploratory analyses reveal significant differences also between the setup and 50% condition and each of the other conditions. **p ≤ 0.005, ***p ≤ 0.001. Mean values and standard error of the means are depicted.

Nonparametric post-hoc testing including correction for multiple testing revealed a significant difference between all four conditions (*Z* ≤ −2.641, *p* ≤ 0.008, *r* ≥ 0.341; Figure 2). On a 9-point Likert scale ranging from 1 to 9 mean estimated external influence (± standard error of mean) amounted to 3.43 ± 0.30 during “setup & confirmation,” to 2.97 ± 0.21 during “setup & 50% condition,” to 2.18 ± 0.19 during “setup & negation,” and to 1.61 ± 0.17 during “no setup & negation.” Our first hypothesis, that the intervention comprising both components (“setup & confirmation”) has an impact on the estimation of external influence in comparison to the absence of both components (“no setup & negation”) was confirmed (*Z* ≤ −5.482, *p* ≤ 0.001, *r* = 0.708; Figure 2).

The second and third hypotheses expected, that each of the two components, i.e., the physical setup and the information would augment the feeling of external influence. The conditions “setup & negation” and “no setup & negation” differed only in the presence of the physical setup, whereas no influence is announced in either condition. Therefore, a difference between these conditions can be attributed to the “impact of setup.” The difference in the estimated external influence was 0.57 ± 0.16 (mean estimated external influence during “setup & negation” 2.18 ± 0.19 *vs.* during “no setup & negation” 1.61 ± 0.17). Nonparametric testing revealed a significant difference between these two conditions (*Z* = −3.847, *p* < 0.001, *r* = 0.497; Figure 2), such that the physical setup augments the feeling of external influence.

The “impact of information” can be extracted by comparing the conditions “setup & confirmation” and “setup & negation,” as they differ just in the information given to the participants about the attempt of external influence. Whereas in the first condition, participants are briefed on an attempt of influence, in the second condition no attempt of influence is proclaimed. The difference in the estimated external influence between these two conditions amounted to 1.25 ± 0.22 (mean estimated external influence during “setup & confirmation” 3.43 ± 0.30) *vs.* during “setup & negation” 2.18 ± 0.19). Nonparametric testing revealed a significant difference between these two conditions (*Z* = −5.218, *p* < 0.001, *r* = 0.674; Figure 2). Thus, the information of attempted influence alone augments the estimated feeling of external influence.

We were therefore able to confirm the second and third hypothesis. The physical setup and the information alone each have a significant impact on external influence estimation.

On an exploratory basis, we added comparisons of the other conditions. They all differed significantly from each other (*Z* ≤ −2.641, *p* ≤ 0.008, *r* ≥ 0.341; Figure 2).

In our sample, we found no significant differences in the estimation of external influence between the three interventions (tDCS, eye contact, and hand touch; *F*(2, 57) = 2.927, *p* = 0.062, partial *ɳ*_p_^2^ = 0.093).

In summary, the estimation of external influence can clearly be modulated by the different conditions. Both components setup and information contribute significantly to this effect. In our sample, the three interventions, i.e., tDCS, eye contact, and hand touch, did not significantly differ in the average estimation of external influence. But the significant interaction of condition and setup points towards a difference in the potential to modulate the estimated external influence.

### Comparison of “impact of setup” and “impact of information”

**Figure 3:**
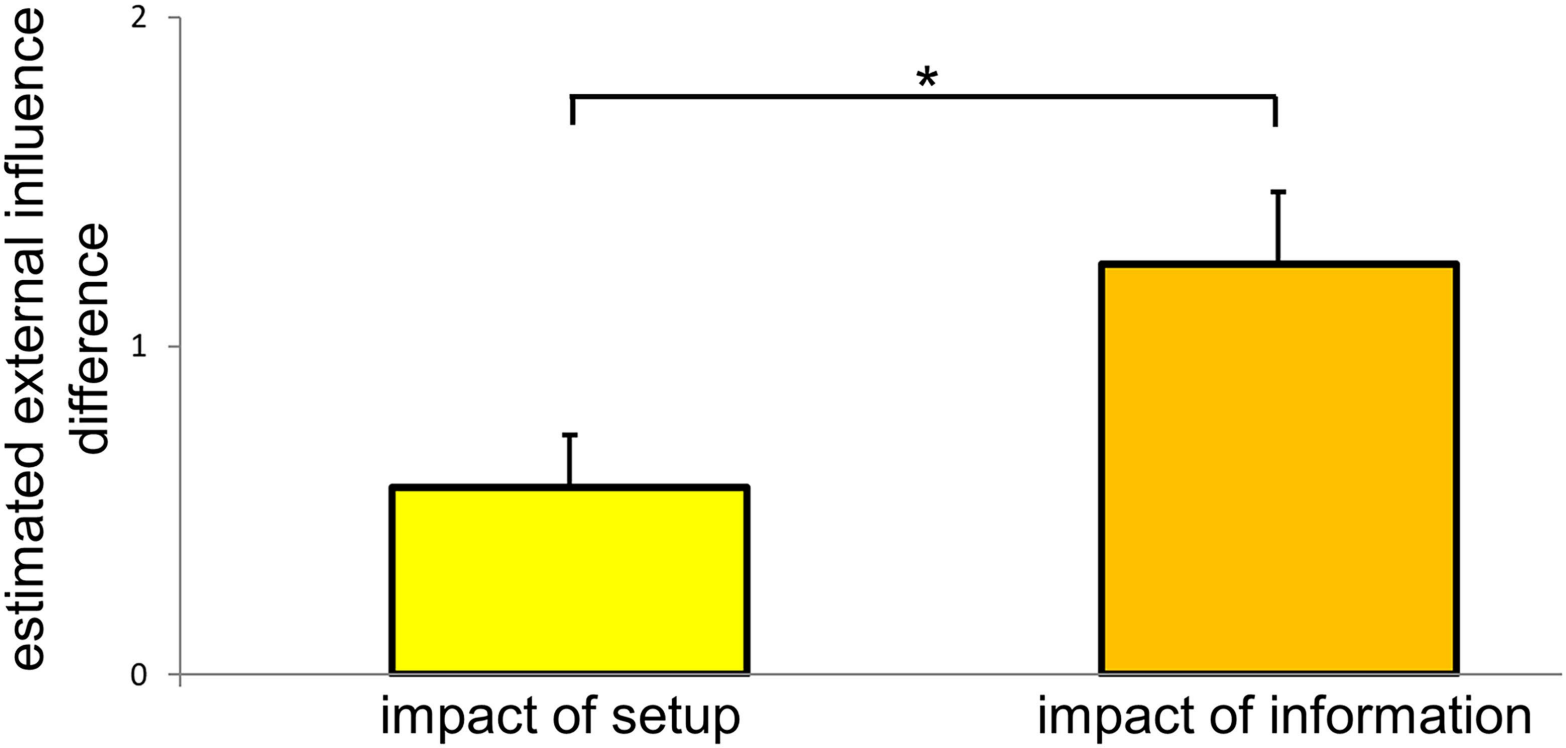
Comparison of “impact of setup” and “impact of information.” “Impact of setup” is the difference of the estimated external influence between the conditions “setup & negation” and “no setup & negation,” which differ in the presence and absence of the setup, respectively. “Impact of information” represents the difference between the estimation of external influence in the condition “setup & confirmation” minus “setup & negation.” In the first condition participants were informed that external influence is attempted (confirmation), whereas they were informed that no external influence is attempted (negation) in the second condition. *p < 0.05. Mean values and standard error of the means are depicted.

Additional explorative analyses were added to compare the “impact of setup” with the “impact of information.” The “impact of information” significantly outperformed the “impact of setup” concerning the extent of estimated external influence (*Z* = −2.394, *p* = 0.016, *r* = 0.310; Figure 3).

### External influence induction comparing the different setups

Mixed ANOVA revealed a significant interaction of condition by setup [*F*(4.415, 125.816) = 3.416, *p* = 0.009, partial *ɳ*_p_^2^ = 0.107]. Nonparametric post-hoc tests including correction for multiple testing confirmed for the **tDCS** condition a significant difference between “setup & confirmation” and “setup & negation” (*Z* = −3.243, *p* < 0.001, *r* = 0.725), between “setup & confirmation” and “no setup & negation” (*Z* = −3.310, *p* < 0.001, *r* = 0.740), between “setup & negation” and “setup & 50% condition” (*Z* = −3.078, *p* < 0.001, *r* = 0.688), and between “setup & 50% condition” and “no setup & negation” (*Z* = −2.858, *p* = 0.003, *r* = 0.639). Thus, for the setup tDCS, the first hypothesis, that the entire intervention combining setup and information has a significant input on external influence estimation, as well as the third hypothesis, that the information alone has an impact, were corroborated.

For the intervention **eye contact,** all four conditions differed significantly from another (*Z* ≤ −2.854, *p* ≤ 0.003, *r* ≥ 0.638). Thus, for the setup eye contact, our three hypotheses, that the whole intervention as well as the setup only and the information only have a significant influence on external influence estimation were corroborated.

For the **hand touch** intervention there was a difference between “setup & confirmation” and “setup & negation” (*Z* = −2.160, *p* = 0.029, *r* = 0.483) and between “setup & confirmation” and “no setup & negation” (*Z* = −2.292, *p* = 0.020, *r* = 0.513), between “setup & negation” and “setup & 50% condition” (*Z* = −3.409, *p* < 0.001, *r* = 0.762), and between “setup & 50% condition” and “no setup & negation” (*Z* = −3.409, *p* < 0.001, *r* = 0.762). Similar as to the setup tDCS our first and third hypotheses were also confirmed for the setup hand touch.

Summarizing the results, for all three setups we found a significant effect of the entire intervention (including setup and information) as well as for the impact of information, whereas the impact of setup was only significant for the eye contact setup.

**Figure 4:**
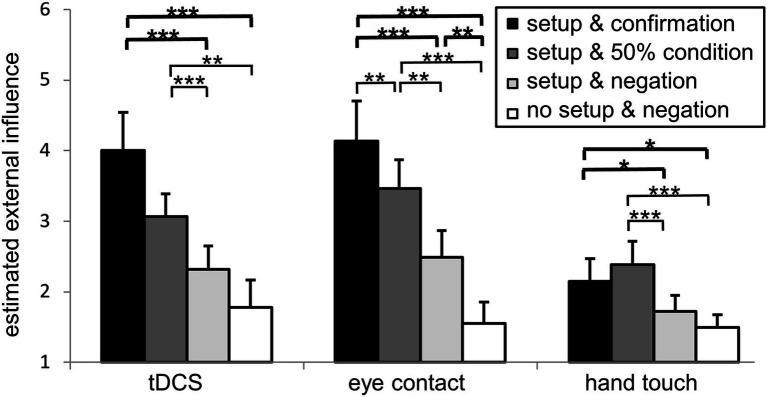
External influence estimation comparing the different setups and conditions. Effect of condition separately per setup. “Setup & confirmation” (black), “setup & 50% condition” (dark grey), “setup & negation” (light grey) and “no setup & negation” (white) are given for tDCS, eye contact, and hand touch. Results of hypothesis driven analyses comparing the conditions are marked with bold lines and asterisks above, exploratory analyses in slim lines and asterisks below. *p ≤ 0.05, **p ≤ 0.005, ***p ≤ 0.001. Mean values and standard error of the means are depicted.

A graphical representation is given in Figure 4.

### External influence induction during the “setup & 50% condition”

In the “setup & 50% condition” the participants judged the attempt of influence correctly in about half of the trials (tDCS 0.51 ± 0.09, eye contact 0.47 ± 0.07, hand touch 0.51 ± 0.09, all interventions 0.50 ± 0.09). The accuracy rate did not differ statistically significantly from the chance level probability of 0.5 (*p* = 0.95).

Comparing trials of the **50% condition with “attempt” and “no attempt”** of influence, no difference in the estimation of external influence was found [*F* (1, 57) = 1.928, *p* = 0.170, partial *ɳ*_p_^2^ = 0.033] as well as no difference between the setups [*F*(2, 57) = 2.301), *p* = 0.109, partial *ɳ*_p_^2^ = 0.075]. The number of trials with “attempt” and “no attempt” was fixed to 12 trials each (see Materials and Methods section). Mean estimated external influence was 3.02 ± 0.21 in the condition “setup & attempt” and 2.93 ± 0.22 in the condition “setup & no attempt.”

**Figure 5:**
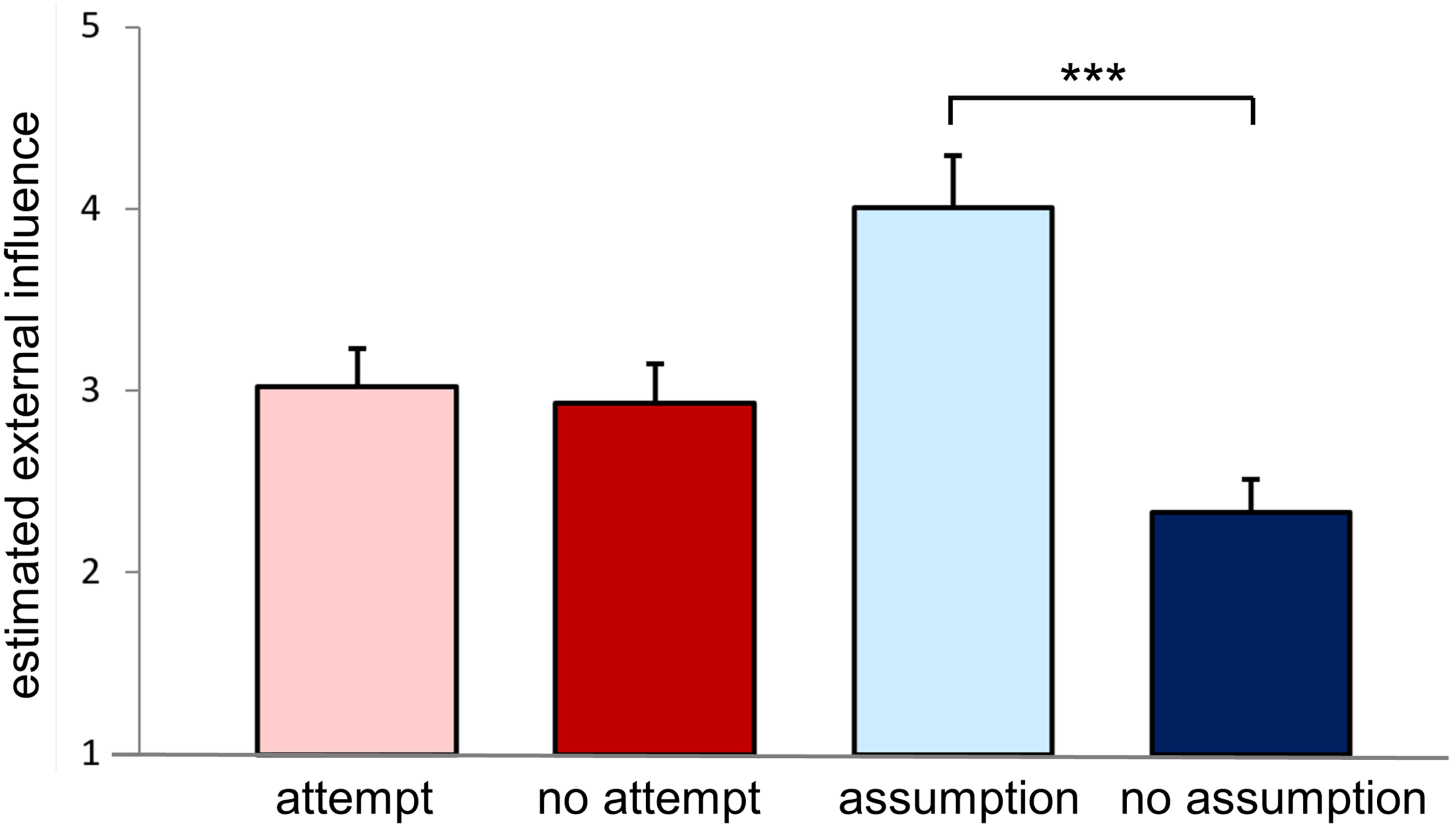
External influence estimation during the “setup & 50% condition.” During the “setup & 50% condition” participants were informed that external influence will be attempted in 50% of the trials but no information was provided regarding each specific trial. 24 trials were carried out. The bar graph shows estimated external influence depending on interventional condition [trials with “attempt” of external influence (light red) *vs.* trials with “no attempt” of external influence (dark red)] and responses of the participants [“assumption” of external influence (light blue) vs. “no assumption” of external influence (dark blue)]. It should be noted that the number of trials was fixed regarding interventional conditions (*n* = 12 attempt, *n* = 12 no attempt), whereas assumptions of external influence varied considerably between subjects [*n* = 0–15 assumption (mean 10.0 ± 4.1), *n* = 9–24 no assumption (mean 14.0 ± 4.1)]. ***p ≤ 0.001. Mean values and standard error of the means are depicted.

Dividing the **50% condition in those trials with “assumption” and “no assumption”** of influence, in the trials with assumed influence the estimated external influence was significantly higher than in trials with no assumed influence [*F*(1, 51) = 56.912, *p* < 0.001, partial *ɳ*_p_^2^ = 0.527]. Mean estimated external influence was 4.01 ± 0.28 in the condition “setup & assumption” and 2.33 ± 0.18 in the condition “setup & no assumption.” There was no significant difference between the setups [*F*(2, 51) = 1.669, *p* = 0.193, partial *ɳ*_p_^2^ = 0.062; Figure 5]. The number of trials, in which the participants confirmed and denied an influence (“assumption” and “no assumption”), respectively, varied between participants between zero trials with assumed influence to 15 trials with assumed influence (mean 10.0 ± 4.1 trials with assumed influence). Inversely, the participants assumed no influence in 9 to 24 trial during this condition (mean 14.0 ± 4.1 trials with no assumed influence).

The results in the “setup & 50% condition” confirm the blinding of the participants concerning the current attempt of influence, whereas the results comparing the different judgement of the participants validate the perceived modulation of external influence.

### Relation of the estimated external influence with response latency

**Figure 6:**
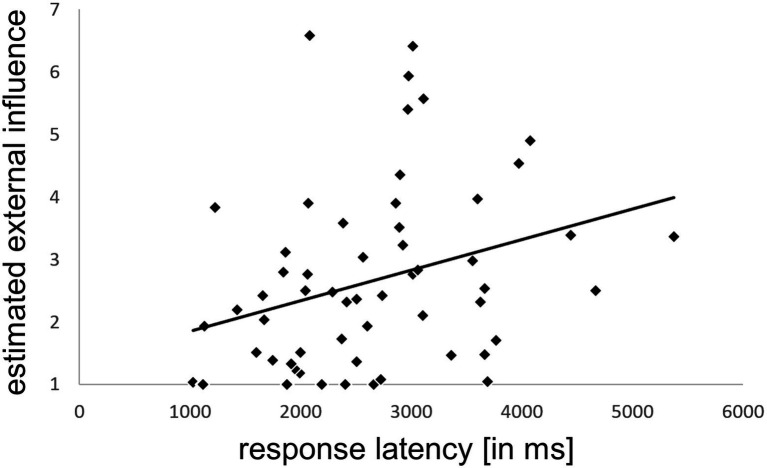
Correlation of the estimated external influence with response latency. The average external influence rating per participant correlated significantly with the corresponding average response latency for estimating this influence (across all four conditions). Due to non-normally distributed data nonparametric testing was performed (*r*_S_ = 0.392, *p* = 0.002). ms = milliseconds.

The average level of estimated external influence (across all conditions) per participant correlated significantly with their corresponding average response latency for estimating this influence (*r*_S_ = 0.392, *p* = 0.002, Figure 6).

**Figure 7:**
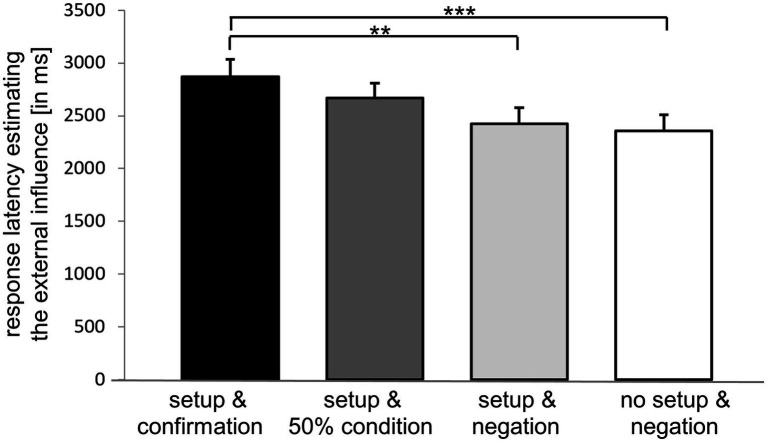
Response latencies for estimating the external influence. Conditions are color-coded using “setup & confirmation” (black), “setup & 50% condition” (dark grey), “setup & negation” (light grey) and “no setup & negation” (white). **p ≤ 0.005, ***p ≤ 0.001. Mean values and standard error of the means are given.

Comparing mean response latencies for assessing external influence during different conditions and setups, we identified a significant main effect of the condition [*F* (2.733, 155.803) = 5.276, *p* = 0.002, partial *ɳ*_p_^2^ = 0.085], which was confirmed in post-hoc nonparametric tests between “setup & confirmation” and “setup & negation” (*Z* = −2.878, *p* = 0.004, *r* = 0.372) as well as between “setup & confirmation” and “no setup & negation” (*Z* = −3.850, *p* < 0.001, *r* = 0.497; Figure 7). Mean response latency (± standard error of mean) for estimating the external influence was 2,870 ± 163 ms for “setup & confirmation,” 2,667 ± 142 ms for “setup & 50% condition, 2,422 ± 152 ms for “setup & negation,” and 2,356 ± 156 ms for “no setup & negation.”

### Effects of object categories on induced feelings of external influence, intensity and valence of imaginations

Additional analyses addressed the question of whether the given object categories (general, specific positive, specific negative) had a significant impact on the estimation of external influence, intensity or valence of the imagination. There was a statistically significant effect of object categories on the emotional valence such that specific negative objects had a lower emotional valence in comparison to specific positive and to general objects. The detailed results can be found in the Supplementary material.

### Correlation of the estimation of external influence with crystallized intelligence and psychopathological measures

Additional analyses of the correlation of the estimation of external influence with crystallized intelligence and psychopathological measures can be found in the Supplementary material.

## Discussion

This study aimed to investigate subjective feelings of influence in healthy individuals. For this purpose, we established an experimental approach to induce feelings of external influence in a visual imagery task. The intervention consisted 1) of an information, whether an influence was attempted or not, and 2) of a physical setup to perform this influence (tDCS stimulation, eye contact, hand touch).

We found a statistically significant main effect for the condition. Our first hypothesis, that the intervention combining the physical setup as well as the information of an attempt of external influence augments the feeling in comparison to the absence of setup and information, was confirmed. We were also able to show that the experimental setup alone as well as the information alone was sufficient to increase the estimated external influence significantly, so the second and third hypotheses were therefore also verified. Thus, the current intervention is able to modulate the feeling of external influence. The average estimation of external influence did not significantly differ across the different setups, but the interaction of condition and setup was significant. The dimension of the amplification concerning the estimation of external influence was similar for tDCS and eye contact and ranged between a more than twofold increase in the “setup & confirmation” condition compared to the “no setup & negation” condition, and approximately an 1.7-fold increase of the “setup & confirmation” condition in comparison to the “setup & negation” condition. The enhancement for the hand touch condition shows a 1.4-fold and 1.3-fold increase, respectively. These results suggest that the three setups differ in their potential to evoke the feeling of external influence.

Comparing the contribution of the setup and the information given to the participants, the “impact of information” had a significantly larger effect on the modulation of the feeling of external influence than the “impact of setup.” Thus, it is especially the information given to the participants and the resulting expectation that drives the estimation of external influence, more strongly than the physical setup per se.

The results during the “setup & 50% condition” validated the blinding of the participants concerning the attempt of external influence: The accuracy rate of correctly assumed attempts of external influence did not significantly differ from chance level. Furthermore, the rating of the external influence did not differ between trials with an “attempt” and “no attempt” of influence. As intended by the experimental design, the judgement of the participants concerning attempted influence in a trial and the rating of the external influence during that trial were related.

The absolute maximum mean values for the estimation of external influence ranged around 4 on a Likert scale from 1 to 9, so the intervention seems to have medium effects in healthy controls. This indicates that the intervention is powerful enough to provoke changes in healthy controls while not reaching a ceiling effect. Thus, it is likely that more intense experiences of external influence – as they might be expected in psychotic patients – can still be measured.

Klock et al. (2021) developed a related paradigm to modulate the authorship of thoughts. In our paradigm we varied the given stimuli using different general and specific categories and additionally varying the emotional valence of the stimuli. Moreover, we established different conditions to decipher the influence of the experimental setup and the information given to the participants, in the design of Klock et al. these two components could not be differentiated. We also used different experimental setups, one with a technical device and two with social signals. We were therefore able to show, that different setups can modulate the feeling of external influence. We made sure, that we give the participants no misleading information (i.e., the device would be active when it was not). And lastly, in our study, we focused solely on the aspect of external influence modulation during instructed imaginations in healthy individuals as from our point of view “feelings of external influence” and “feelings of own authorship” do not mandatorily directly correlate in every setting.

For the construct of authorship or “sense of agency,” e.g., the phenomenon of feeling or acting as a self or in other words the ascription of actions or thoughts to oneself vs. to another person, several similar, but not fully comparable constructs have been described in the literature, (Haggard, 2017; Grunbaum and Christensen, 2020). The model of sense of agency has been split up in further detail in a 2-by-2 construct (Grunbaum and Christensen, 2020): 1) the type of awareness differentiates between the “ability sense of agency” as a cognitive function and the “phenomenal character sense of agency” as a direct neuronal feedback on a stimulus, and 2) the effect can be evaluated either as body movement itself (action), e.g., a keypress, or as change in the environment (effect), e.g., a sound following a keypress.

Additionally, the measurement of the sense of agency differs using either implicit measures like intentional binding, i.e., the temporal convergence of a voluntary action and a sensory consequence (Haggard et al., 2002; Moore and Obhi, 2012), or explicit measures, i.e., the question, whether or not an event was self-generated. Temporal binding is stronger in conditions focusing on the effect of one’s actions, whereas agency ratings respond stronger to the executed action. It is crucial to consider, which output measure is used, as both seem to reflect different processes and do not necessarily correlate (Schwarz et al., 2019).

Experimental setups assessing sense of agency focus almost exclusively on the phenomenal character sense of agency, i.e., manipulations of motor-sensory corollary loops and questions concerning self-other-distinction. Participants get temporal or regional distorted of feedback of own movements (Engbert et al., 2008; Synofzik et al., 2010; Timm et al., 2014; Haering and Kiesel, 2015) and given information concerning the personal influence (Moore et al., 2009; Desantis et al., 2016). Further experimental settings comprised active vs. passive movements and display of one’s own vs. someone else’s hand (Uhlmann et al., 2021).

As we did not assess markers of sense of agency in our study, we can only speculate about the relationship between the feeling of external influence in our paradigm and sense of agency markers. Our assessment method of asking the participants for their estimation of external influence appears conceptually similar to the explicit measure of sense of agency. In particular, a negative association between the estimated feeling of external influence and the conceptualization of sense of agency as “feeling or acting as a self” could be expected. But also, a view of complementary and independent concepts is conceivable. A person stably (not) assessing his-or herself as the author of thoughts or acts could vary in his/her estimation of the level of external influence during the different conditions of our experiment. In this scenario, the sense of agency stays the same while the subjective level of external influence changes.

The neuronal basis of sense of agency in motor tasks is explained with a self-generated motor action, that is accompanied by an efference copy of the motor command (corollary discharge) and a sensory feedback. Both inputs are compared using predictive and retrospective processes. If they fit together, an internal authorship is assumed. If they diverge, the event is interpreted as externally caused (Frith et al., 2000b; Frith, 2012). Therefore, source monitoring deficits, i.e., difficulties “distinguishing between the origin of endogenous (i.e., internally or self-generated) and exogenous (i.e., externally or other-generated) stimuli” lead to impaired sense of agency (Nelson et al., 2014a,b). In broader theoretical approaches, beneath the sense of agency for actions also a sense of authorship of thoughts has been carved out building a multilayered “sense of agency” concept with both motor-sensory corollaries and mental thoughts, beliefs and intentions (Synofzik et al., 2008). The theoretical concept seems very plausible, but has to date scarcely been addressed in experimental setups. Concerning this aspect, our paradigm takes a novel approach, as our outcome measure is neither a motor response nor a visual or auditory effect, but the assumed external influence on an instructed imagination. It is therefore more similar to the aforementioned authorship of thoughts, even if the exact relationship between both constructs remains unanswered at the moment.

In the literature on imagination, it has been shown that perception and imagination share broad common, especially posterior, networks and that activation is correlated with vividness of imagery (Ishai et al., 2000; Dijkstra et al., 2017; Fulford et al., 2018; Dijkstra et al., 2019). In the study at hand, we used a visual text cue to instruct the imagination, achieving a stable performance of imaginations with high intensities, i.e., a mean score of 6.6 ± 1.3 on a 9-point Likert scale, similar across conditions and interventions. Thus, differences estimating the external influence cannot just be attributed to differences in imagination intensities. We also controlled for the emotional content of the imagery. All conditions revealed similar levels of emotional valence in the slightly positive range around 6.0 ± 0.9 on a 9-point Likert scale (from 1 = very unpleasant to 9 = very pleasant). This means that the estimation of external influence seems not primarily driven by variations of the emotional valence.

Previous work on the sense of agency (mainly of actions) showed that the sense of agency can be modified. Experimental settings to modify sense of agency included manipulations of video feedback concerning own hand movements by varying the time or the exact direction of the movement (Engbert et al., 2008; Synofzik et al., 2010; Timm et al., 2014; Haering and Kiesel, 2015), the amount of physical effort (Demanet et al., 2013), performance (Wen et al., 2017), cognitive effort (Van den Bussche et al., 2020), and given instruction on exertion of agency (Moore et al., 2009; Desantis et al., 2016). Although if the exact relationship between sense of agency and feeling of external influence is not solved yet, we were able to show, that the feeling of external influence can be modified in healthy individuals by instructions and physical setup. Nevertheless, the results may support the model that the integration of internal and external cues can affect the sense of agency as well as the estimation of external influence. And the results from different experimental setups—as already shown in the literature as well as with our paradigm – may support the view of a continuum of authorship/agency ranging from full control to complete external origin with all shades in between.

In our paradigm, the estimated level of external influence correlated with participants’ mean response latency for assessing this influence. Longer average response latencies were associated with higher average estimations of external influence or – in other words – persons who tended to decide fast rated the perceived external influence low. As we do not know about the direction of the correlation, one could also state, that persons who perceived the external influence as low tended to rate this influence faster. We found an approximately linear relation without saturation effect. This connection is supported by previous results in a sense of agency paradigm. Participants viewed an image of their hand either in real time or with a delay while it was brushed, passively moved or actively moved during a so-called induction period of 60 s (Longo and Haggard, 2009). Sense of agency during the induction period was measured using a subjective report questionnaire. Significant levels of agency emerged only following real time feedback of the active movement, but not following the other conditions. The induction period was succeeded by experimental trials during which participants were asked to press a button as quickly as possible in response to a visual stimulus. A higher sense of agency correlated significantly with shorter reaction times (Longo and Haggard, 2009). A difference in this paradigm in comparison to our paradigm, however, concerns the instruction. Whereas in the cited study reaction was instructed to be given as quickly as possible, no timely specification was given in the current study. But in both paradigms, the feeling of agency and lower levels of estimated external influence, respectively, are accompanied by shorter response times. The direction of effect, i.e., whether the feeling of external influence alters the response latency or whether longer times for consideration predict higher levels of estimated external influence, remains an open issue at the moment.

Comparisons between the different conditions in our paradigm unraveled the components that triggered the effects on response latencies. Response latencies are prolonged by confirmation with no further effect of the combination with setup, thus, it seems to be mainly the information given to the participants at the beginning of each condition that interact with response latencies.

As these results are novel and cannot directly be compared to existing knowledge, the results have to be interpreted with caution. But they may indicate an additional cerebral process of estimating the amount of influence taking additional time during our paradigm.

We did not find a correlation of the feeling of external influence assessed in our paradigm with participants’ attitudes concerning supernatural beliefs, proneness to hallucinations or attributional styles (for a more detailed discussion of the results see Supplementary material). This was somehow surprising, as we would assume psychological similarities between these different concepts and towards psychopathological phenomena, especially delusions and delusions of influence. The missing relationship of our paradigm with attitudes concerning supernatural beliefs, proneness to hallucinations or attributional styles may be explained by the selected and uniform sample of participants with therefore small variation and extent of these experiences. We therefore are interested in examining further participant samples with higher variability of the mentioned characteristics and patients’ samples with clear delusions of influence.

In a next step we want to correlate the feeling of external influence assessed in our paradigm with further constructs such as trait markers, fantasy-proneness and susceptibility to further implement the phenomenon in the preexisting psychopathological concepts. Another further project concerns the addition of neurobiological methods to uncover underlying mechanisms. Finally, thinking one more step ahead, the paradigm could not only be used for diagnostic but also therapeutic purposes, as it can open doors for a nuanced consideration of the issue. Participants can experience the variability of the estimation of external influence by different factors (information and setup) and therefore reconsider own judgements. The paradigm could therefore encourage metacognition of alienation experiences.

Regarding the limitations of the current study it should be noted that the generalizability of our findings could be restricted by the characteristics of the study population. Due to recruiting the participants from the degree program “Psychology” of the University of Tübingen, age as well as gender distribution and educational level differ from normal population. Another point concerns the fact that in addition to the different setups (tDCS, eye contact, hand touch) and levels of information about the intended intervention (confirmation, negation), all trials were conducted with an explicit instruction to imagine a specific object. This aspect of the task could already affect participants’ feeling of external influence. In future studies it would be highly interesting to evaluate the extent to which the procedure to evoke imaginations might influence the feelings of external influence. The “degrees of freedom” might be varied systematically (e.g., from specific objects to “any object” or “any imagination”) and to test other forms of stimulus presentation to guide participants’ imagination, e.g., pictures or vocal sounds. In addition, instructions could be extended by imaging not only static objects, but also moving images or sceneries. A variety of more complex stimuli is closer to reality. Scene imagery has been shown to involve further regions including hippocampus and ventromedial prefrontal cortex (Barry et al., 2019), giving further insight in different neural mechanisms of imagination. Finally, data concerning additional possible influencing factors such as suggestibility, personality traits and fantasy-proneness were not collected in the present study and should be taken into consideration in further studies.

In summary the results indicate that it is possible to induce feelings of external influence during instructed imaginations through specific interventions (using physical setup and information) in healthy persons. Since the information about the intended influence turned out to have a stronger impact on feelings of external influence as compared to their physical setup, future studies might focus more strongly on information provision. The techniques applied in healthy persons should be translated to a clinical setting to evaluate if they allow for a quantitative assessment of feelings of external influence in persons experiencing spontaneous delusions of influence due to psychiatric disorders.

## Data availability statement

The raw data supporting the conclusions of this article will be made available by the authors, without undue reservation.

## Ethics statement

The studies involving human participants were reviewed and approved by Ethics Committee of the Faculty of Medicine of the Eberhard Karls University and the University Hospital Tübingen. The patients/participants provided their written informed consent to participate in this study. The individual(s) provided their written informed consent for the publication of any identifiable images or data presented in this article.

## Author contributions

DW, KE, and DR conceptualized the study. NZ, SoP, ShP, MH, DR, and KE performed the data aquisition. MH, DR, NZ, SoP, ShP, KE, AM, and DW analyzed and interpreted the data. KE, DW, and AM wrote the first draft of the manuscript and prepared the figures. All authors contributed to the article and approved the submitted version.

## Funding

This work was supported by the Clinician Scientist program of the University of Tübingen to KNE (Grant number 367-0-0). We acknowledge support by Open Access Publishing Fund of University of Tübingen. The funders had no role in study design, data collection and analysis, decision to publish, or preparation of the manuscript.

## Conflict of interest

The authors declare that the research was conducted in the absence of any commercial or financial relationships that could be construed as a potential conflict of interest.

## Publisher’s note

All claims expressed in this article are solely those of the authors and do not necessarily represent those of their affiliated organizations, or those of the publisher, the editors and the reviewers. Any product that may be evaluated in this article, or claim that may be made by its manufacturer, is not guaranteed or endorsed by the publisher.
