# Supplementary material for: Induced feelings of external influence during instructed imaginations in healthy subjects
Source: Front Psychol. 2022 Nov 1;13:1005479. doi: 10.3389/fpsyg.2022.1005479 (PMC9664387; doi:10.3389/fpsyg.2022.1005479)
Supplement: Supplementary file 1 [file Data_Sheet_1.pdf]

Supplementary Material

1 Supplementary Table 1. Psychometric data of the 60 healthy participants.

	tDCS (n = 20)	Eye contact (n = 20)	Hand touch (n = 20)	P value
IQ	108.3 ± 9.9	107.5 ± 14.5	106.0 ± 8.1	0.803
LSHS-R	8.2 ±5.0	10.2 ± 7.4	9.1 ± 6.1	0.587
SBS	-1.0 ± 2.2	-1.0 ± 2.2	-1.4 ± 1.9	0.745
TAS	48.8 ± 24.2	43.0 ± 23.8	51.8 ± 16.7	0.441
ASF-E positive internal	77.4 ± 9.2	75.3 ± 9.8	78.7 ± 13.5	0.617
ASF-E positive stable	82.9 ± 8.1	75.9 ± 8.4	80.0 ± 8.4	**0.034***
ASF-E positive global	78.7 ± 13.3	81.1 ± 11.5	79.2 ± 11.2	0.794
ASF-E negative internal	64.7 ± 12.9	63.5 ± 9.1	68.3 ± 10.6	0.365
ASF-E negative stable	60.7 ± 13.7	58.6 ± 14.2	46.7 ± 12.8	0.362
ASF-E negative global	51.1 ±15.1	55.3 ±15.7	60.4 ± 16.2	0.177

tDCS = transcranial direct current stimulation; IQ = intelligence quotient; LSHS-R = Launay-Slade hallucination scale – revised version; SBS = Supernatural Belief Scale; TAS = Tellegen Absorption Scale; ASF-E = Attributionsstilfragebogen für Erwachsene. Numbers indicate mean and standard deviation. * $p < 0.05$.

ANOVA analysis revealed no significant differences between the groups concerning intelligence quotient (IQ), proneness to hallucinations (LSHS-R), supernatural beliefs (SBS), or absorption (TAS) (all $F(2, 57) \leq 0.83$, all $p \geq 0.441$). The only difference between groups concerned the ASF-E stable score for positive events. Results were corroborated by Kruskal-Wallis analysis and post-hoc nonparametric testing with Bonferroni correction, which revealed a difference between the tDCS and eye contact setup ($Z = -2.465$, $p = 0.038$, $r = .390$).

Supplementary Table 2. Objects for imagination task.

**General objects (general)**	**Specific objects with a more positive connotation (specific positive)**	**Specific objects with a more negative connotation (specific negative)**
Tier (Animal)	Hase (Rabbit)	Spinne (Spider)
Pflanze (Plant)	Rose (Rose)	Distel (Thistle)
Gebäude (Building)	Palast (Palace)	Ruine (Ruin)
Werkzeug (Tool)	Hammer (Hammer)	Zange (Tongs)
Flüssigkeit (Liquid)	Orangensaft (Orange juice)	Hustensaft (Cough syrup)
Möbel (Furniture)	Sessel (Armchair)	Hocker (Stool)
Gemüse (Vegetables)	Paprika ((Sweet) pepper)	Rosenkohl (Brussel sprouts)
Frucht (Fruit)	Erdbeere (Strawberry)	Zitrone (Lemon)
Kleidungsstück (Piece of clothing)	Handschuh (Glove)	Socke (Sock)
Straße (Street)	Allee (Boulevard)	Gasse (Alleyway)
Elektrogerät (Electrical device)	Fernseher (Television)	Radio (Radio)
Geschäft (Shop)	Feinkostladen (Delicatessen store)	Bahnhofskiosk (Train station kiosk)
Backzutat (Baking ingredient)	Zucker (Sugar)	Mehl (Flour)
Behälter (Container)	Handtasche (Handbag)	Plastiktüte (Plastic bag)
Heißgetränk (Hot beverage)	Kakao (Hot chocolate)	Ingwertee (Ginger tea)
Kosmektikartikel (Cosmetic product)	Parfüm (Perfume)	Pinzette (Tweezers)

Fahrzeug (Vehicle)	Limousine (Sedan)	LKW (Semi-trailer)
Schiff (Ship)	Segelboot (Sailing boat)	U-Boot (Submarine)
Musikinstrument (Musical instrument)	Gitarre (Guitar)	Flöte (Flute)
Gewürz (Spice)	Vanilleschote (Vanilla bean)	Chilischote (Chili)

The participants were asked to imagine 60 different objects. The objects were grouped into three categories: general objects, specific objects with a more positive connotation and specific objects with a more negative connotation. The order was pseudorandomized, so the same category was never presented twice in a row.

3 Supplementary Figure 1. Photos illustrating the three experimental conditions.

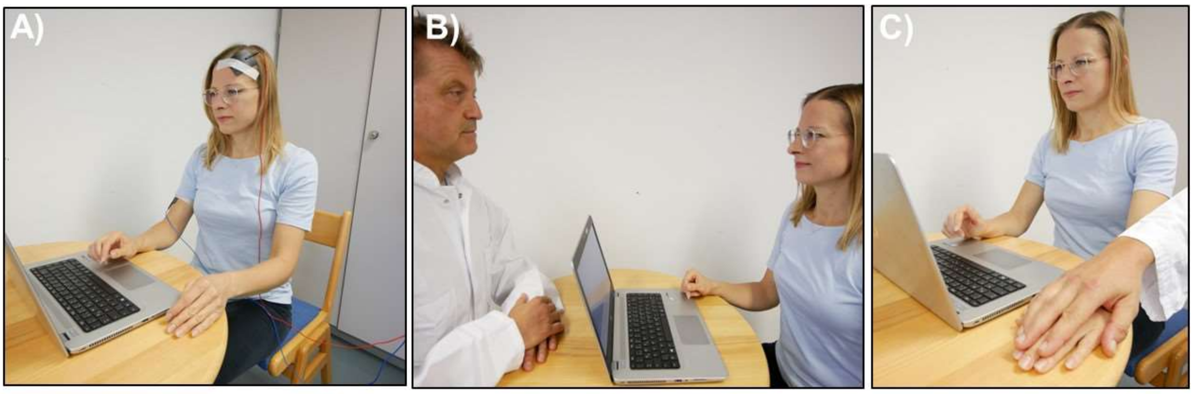

(A) shows the tDCS, (B) the eye contact, and (C) the hand touch condition. During the actual trials no photographs were taken. So, the experimental arrangement was reconstructed for publication purposes. The pictured people are staff members of the Department for Psychiatry and Psychotherapy.

4 Effects of object categories on the emotional valence, the intensity and the estimation of external influence during imagination

The objects to be imagined were classified into three groups concerning the emotional valence: general objects, specific objects with a more positive connotation and specific objects with a more negative connotation.

First, we analyzed whether the implicated difference in emotional content of the objects was actually present in the current participant sample. Indeed, the emotional valence of imaginations differed considerably between the object categories ($F(1.617, 92.164) = 86.060$, $p < 0.001$, partial $\eta_p^2 = .602$). Post-hoc tests revealed significant differences between global and specific negative objects ($Z = -6.530$, $p < 0.001$, $r = .843$) as well as between specific positive and specific negative objects ($Z = -6.365$, $p < 0.001$, $r = .822$). The average ratings were 6.22 ± 0.10 for global objects, 6.3 ± 0.10 for specific positive objects, and 5.29 ± 0.11

for specific negative objects on a 9-point Likert scale ranging from 1 = very low/unpleasant to 9 = very high/pleasant (mean ± standard error of the mean).

Concerning the intensity of imaginations a slight difference was found ($F(1.890, 107.714) = 6.975$, $p = 0.002$, partial $\eta_p^2 = .109$). Nonparametric post-hoc tests uncovered significant differences between the categories global and specific positive ($Z = -2.994$, $p = 0.002$, $r = .387$) as well as between global and specific negative objects ($Z = -3.979$, $p < 0.001$, $r = .514$). The average ratings were 6.68 ± 0.16 for global objects, 6.47 ± 0.15 for specific positive objects, and 6.38 ± 0.14 for specific negative objects (mean ± standard error of the mean), with higher ratings indicating more intense imaginations.

The induced feeling of external influence did not differ between object categories (general objects, specific positive objects, specific negative objects) ($F(1.694, 96.570) = 2.832$, $p = 0.072$, partial $\eta_p^2 = .047$). The ratings of estimated external influence were 2.53 ± 0.18 for global objects, 2.71 ± 0.20 for specific positive objects, 2.64 ± 0.19 for specific negative objects (mean ± standard error of the mean).

A graphical representation of the effects of object categories on emotional valence, intensity and induced feeling of external influence during imagination are depicted in Supplementary Fig. 2 A, B, C.

Putting the results of object categories in context, the participants confirmed our intended difference with a more negative connotation for the specific negative objects in comparison to both other categories (specific positive and general) in their estimation of emotional valence during the imagination. Interestingly, for the intensity we also found a difference with a more intense imagination for general objects in comparison to both specific categories (specific positive and specific negative objects). From these results it can be speculated that general categories, giving more opportunity for own ideas and imagination, lead to a more intense experience of imagination. Lastly, the object categories do not modulate the estimated external influence during the imagination. This finding provides evidence of generalizability of the results and refutes the possibility that the observed differences in the external influence estimation are based on differences in emotional valence content.

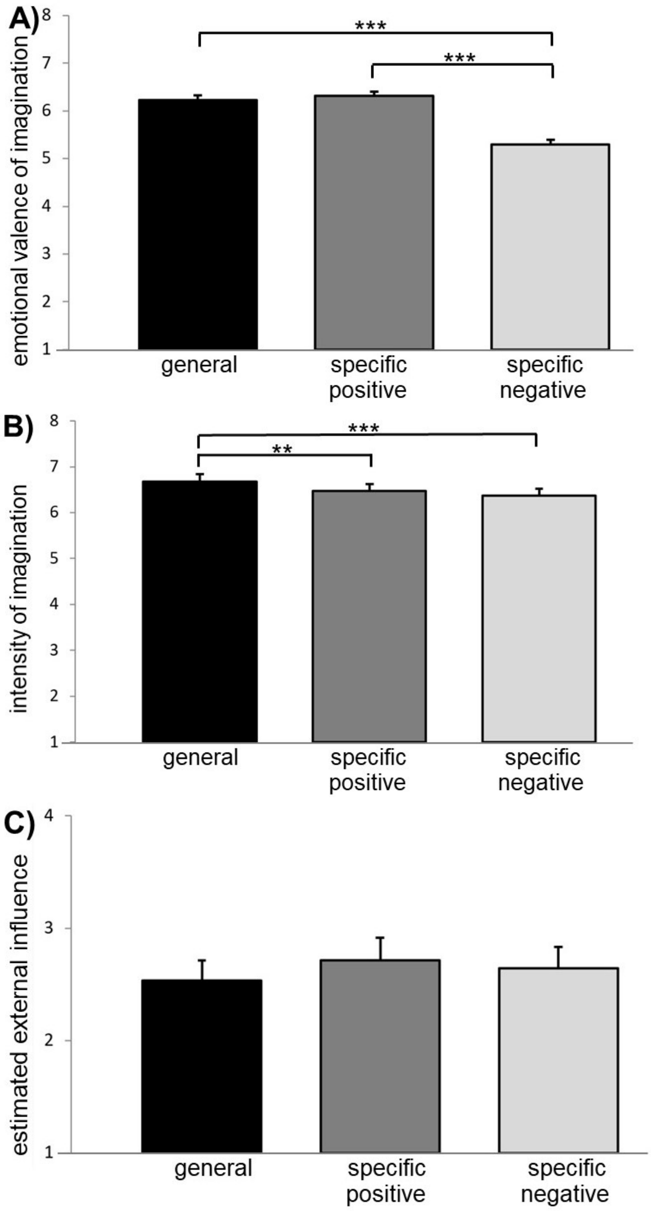

Supplementary Figure 2. Emotional valence, intensity and estimated external influence for the different object categories (general, specific positive, specific negative).

(A) The emotional valence of imagination was significantly lower for specific negative objects (light gray) in comparison to general objects (black) as well as specific positive objects (dark grey). (B) The intensity of imagination was significantly higher for the category “general” (black) compared to “specific positive” (dark grey) and “specific negative” (light grey). (C) Induced feeling of external influence did not differ between the object categories “general” (black), “specific positive” (dark grey) and “specific negative” (light grey). Please consider the different scaling of the y-axis between A/B and C. ** $p \leq 0.005$, *** $p \leq 0.001$. Mean values and standard error of the means are depicted.

5 Effect of condition on intensity and emotional valence judgements

In addition to the estimation of external influence the participants were asked for their judgement of the intensity and the emotional valence of the imagination on a 9-point Likert scale ranging from 1 to 9 during each imagination trial.

Applying a mixed ANOVA, the intensity of the imagination differed between the conditions ($F(2.739, 156.120) = 2.860$, $p = 0.043$, partial $\eta_p^2 = .048$), but not between the different setups ($F(2, 57) = 0.131$, $p = 0.877$, partial $\eta_p^2 = .005$). The interaction of condition and setup was also not significant ($F(5.478, 156{,}130) = 0.856$, $p = 0.521$, partial $\eta_p^2 = .029$). The significant effect of the condition was confirmed using the Friedman test. However, post-hoc testing using the Wilcoxon procedure and correcting for multiple testing revealed no significant differences. The mean intensity estimation (± standard error of the mean) for “setup & confirmation” was 6.51 ± 0.17, for “setup & 50% condition” 6.39 ± .15, for “setup & negation” 6.52 ± 0.16, and for “no setup & negation” 6.75 ± 0.18. A graphical representation is found in Supplementary Figure 3A.

The mean estimated intensity of the imagination did not correlate with the corresponding mean estimation of external influence across participants ($r_S = -0.183$, $p = 0.163$).

Regarding the emotional valence judgement a significant main effect of the condition was found ($F(2.714, 154.672) = 3.180$, $p = 0.030$, partial $\eta_p^2 = .053$). Nonparametric post-hoc tests confirmed a statistically significant difference between “setup & confirmation” and “no setup & negation” ($Z = -3.150$, $p = 0.001$, $r = .407$), whereas the other conditions did not significantly differ. There was no significant difference between the setups ($F(2, 57) = 0.315$, $p = 0.731$, partial $\eta_p^2 = .011$) and no significant interaction of condition by setup ($F(5.427, 154.672) = 0.402$, $p = 0.860$, partial $\eta_p^2 = .014$). The mean estimation of emotional valence (± standard error of mean) during “setup & confirmation” was 5.84 ± 0.11, during “setup & 50% condition” 5.89 ± 0.09, during “setup & negation” 5.97 ± 0.18, and during “no setup & negation” 6.11 ± 0.11. A graphical representation can be found in Supplementary Figure 3B.

The emotional valence of the imagination did not correlate with the corresponding estimation of external influence across participant ($r_S = -0.159$, $p = 0.226$).

However, we found a strong correlation of the mean intensity and the mean emotional valence of the imaginations of each participant ($r_S = 0.583$, $p < 0.001$). The scatter plot is depicted in Supplementary Fig. 4. This means that positively connoted objects are imagined with a higher intensity than negatively connoted objects. Similar results have shown that negative emotional processing interferes with concrete imagery (Behar et al, 2012; Stober, 1998).

These complementary results strengthen the validity of our main results and conclusions. They confirm that the intensity of imagination did not differ between conditions, such that neither the setup nor the information nor the combination of both significantly modulated the intensity of the imagination. Further supportive evidence came from the absent correlation of the intensity with the estimated external influence. For the emotional valence we found a slight but significant difference between the condition “setup & confirmation” and “no setup & negation”, so the entire intervention with combination of setup and information seems to have a mild negative impact on the emotional valence of the imagination. One could speculate that the effort of setup and attempted influence together are sufficient to slightly impair the participant’s experienced freedom and therefore also the hedonic appraisal during the imagination. We found no indication for a direct link to the estimation of external influence, as emotional valence and external influence estimation did not correlate. In the literature,

there is some evidence that positive feelings or action outcomes are associated with a more pronounced sense of agency in comparison to negative feelings or outcomes (reviewed in Kaiser et al, 2021). For the problem of the comparison between the estimated external influence assessed in our paradigm versus the concept of sense of agency see discussion section in the main manuscript. The results cannot be generalized without restrictions, as results (e.g. between explicit and implicit sense of agency measurements) are in part contradictory and conditions have to be very accurately controlled as there seems to be a bidirectional correlation of affective processing and sense of agency.

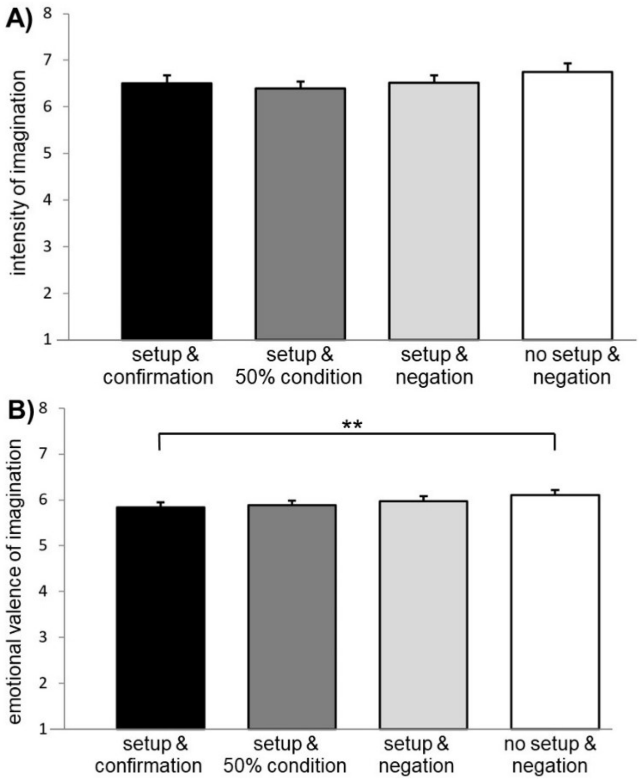

Supplementary Figure 3. Imagination intensity and emotional valence of imagination for the different conditions.

(A) Imagination intensity did not differ between the conditions “setup & confirmation” (black), “setup & 50% condition” (dark grey), “setup & negation” (light grey) and “no setup & negation” (white). (B) The emotional valence of imaginations was slightly lower (less pleasant) during “setup & confirmation” (black) vs. “no setup & negation” (white). ** $p \leq 0.005$. Mean values and standard error of the means are depicted.

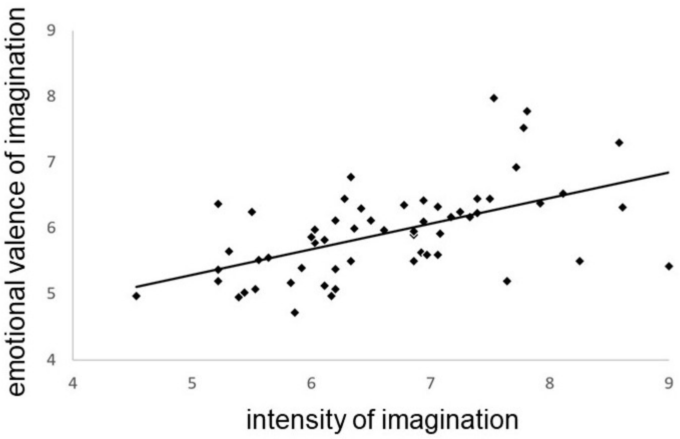

Supplementary Figure 4. Correlation of emotional valence and intensity of imagination.

The average emotional valence and average intensity of imagination per participant (across all four conditions) correlated significantly with more pleasant imaginations rated as being more intense. Due to non-normally distributed data nonparametric testing was performed ($r_S = 0.583$, $p < 0.001$).

6 Correlation of the estimation of external influence with crystallized intelligence

The estimation of external influence did not correlate with crystallized intelligence quotient (IQ), as assessed with the Multiple-choice Vocabulary Intelligence Test (MWT-B), in any condition ($r_S = -0.063$, $p = 0.631$). Thus, differences in the estimation of external influence cannot simply be attributed to differences in intelligence level.

7 Correlation of the estimation of external influence with psychopathological measures

The estimated external influence during the paradigm was correlated to other concepts of delusional and hallucination proneness and attributional style concepts by correlational analyses applying Spearman's rho.

7.1 Questionnaires concerning near psychotic symptoms

There was no significant correlation of the estimation of external influence with proneness to hallucinations (LSHS-R), supernatural beliefs (SBS) as well as absorption (TAS) (Supplementary Table 2).

7.2 Attributional style

The attributional style questionnaire documents the participants' attitude in eight positive and eight negative situations concerning internal causation ("internal"), generalization to other situations ("global") and stability over time ("stable"). There was no significant correlation of the estimation of external influence with attributional style attitudes (Supplementary Table 2).

Supplementary Table 2. Correlations of the estimation of external influence with other constructs.

Score	Mean estimation of external influence
LSHS-R	r_S = .121 / n.s.
SBS	r_S = .191 / n.s.
TAS	r_S = -.008 / n.s.
ASF positive situations internal attribution	r_S = .102 / n.s.
ASF positive situations stable attribution	r_S = .177 / n.s.
ASF positive situations global attribution	r_S = .234 / n.s.
ASF negative situations internal attribution	r_S = -.217 / n.s.
ASF negative situations stable attribution	r_S = .011 / n.s.
ASF negative situations global attribution	r_S = .084 / n.s.

LSHS-R = Launay-Slade hallucination scale – revised version; SBS = Supernatural Belief Scale. TAS = Tellegen Absorption Scale; ASF-E = Attributionsstilfragebogen für Erwachsene. Given are Spearman's Rho correlation coefficient (r_S) and p values. Two-sided testing. P values < 0.05 are considered significant. n.s. = not significant.

8 Discussion

These supplemental analyses concerning the correlation of the estimation of external influence with personal trait markers of attributions and beliefs revealed a negative correlation of the estimation of external influence with the internal attribution style for negative events, i.e. the less events were internally attributed the higher the external influence was rated, and a positive correlation of the estimated external influence with global attribution of positive events, i.e. the more global positive events were rated the higher the external influence was estimated.

The supernatural belief scale (SBS) focusses on universal religious beliefs and therefore correlates with religiosity (Jong, 2013). In our sample we found no association of religiosity with the experience of external influence, so that both paradigms represent different constructs. Jong et al. found a mean SBS for religious people of 1.51 ± 1.79, whereas non-religious people scored -1.15 ± 1.62 and atheists -2.35 ± 1.45 (Jong, 2013). Our sample had a mean SBS value of -1.13 ± 2.1 and therefore lies in the range of the previously described non-religious persons.

The LSHS scale ranges from 0 to 48 points. Previous work showed that healthy persons with high scores in the Launay-Slade hallucination scale (LSHS, mean ± SD 34.8 ± 5.49) were more likely to develop visual and auditory hallucinations in an instructed hallucination procedure than people with low LSHS scores (LSHS mean ± SD 5.2 ± 1.98), whereas the

groups did not differ in terms of suggestibility (Young, 1985). The mean LSHS-R score in our sample amounts 9.2 ± 6.2 points, representing lower levels of proneness to hallucinations. Second, the most decisive difference lies in the task, as our task does not focus on and does not instruct hallucinations but imagery.

Absorption is the tendency to become absorbed or highly involved in sensory or imaginative experiences (Tellegen & Atkinson, 1974). The construct of absorption correlates with hypnotizability (Green & Council, 2004) and higher levels of hypnotizability were accompanied with better visuospatial imagery in a mental rotation task (Incognito et al, 2019). Our task does not require elaborated spatial conception, so the results cannot directly be generalized onto our paradigm.

The estimation of external influence did not correlate with questionnaires regarding supernatural beliefs and proneness to hallucinations and delusions in our sample. It has to be noted however that our sample represents a selected and therefore more uniform group and does not fulfil the criteria of a representative population-based sample with higher variance (see limitations in the main manuscript). Thereby, significant correlations in the general population may be missed.

9 References

Behar, E., McGowan, S. K., McLaughlin, K. A., Borkovec, T. D., Goldwin, M. & Bjorkquist, O. (2012) Concreteness of positive, negative, and neutral repetitive thinking about the future. *Behav Ther*, 43(2), 300-12.

Green, J. P. & Council, J. R. (2004) Does the positive keying of the TAS inflate the absorption-hypnotizability link? *Int J Clin Exp Hypn*, 52(4), 378-88.

Incognito, O., Menardo, E., Di Gruttola, F., Tomaiuolo, F., Sebastiani, L. & Santarcangelo, E. L. (2019) Visuospatial imagery in healthy individuals with different hypnotizability levels. *Neurosci Lett*, 690, 158-161.

Jong, J. B., M.; Halberstadt, J. (2013) Fear of Death and Supernatural Beliefs: Developing a New Supernatural Belief Scale to Test the Relationship. *Eur J Pers*, 27, 495-506.

Kaiser J, Buciuman M, Gigl S, Gentsch A, Schutz-Bosbach S. The Interplay Between Affective Processing and Sense of Agency During Action Regulation: A Review. Front Psychol. 2021;12:716220.

Stober, J. (1998) Worry, problem elaboration and suppression of imagery: the role of concreteness. *Behav Res Ther*, 36(7-8), 751-6.

Tellegen, A. & Atkinson, G. (1974) Openness to absorbing and self-altering experiences ("absorption"), a trait related to hypnotic susceptibility. *J Abnorm Psychol*, 83(3), 268-77.

Young, H. F. B., R. P.; Slade, P. D.; Dewey, M. E. (1985) The role of brief instructions and suggestibility in the elicitation of auditory and visual hallucinations in normal and psychiatric subjects. *The Journal of Nervous and Mental Disease*, 175, 41-48.
